# A cross-sectional study on nutrient intake and -status in inflammatory bowel disease patients

**DOI:** 10.1186/s12937-016-0178-5

**Published:** 2016-06-08

**Authors:** Jona B. Vidarsdottir, Sigridur E. Johannsdottir, Inga Thorsdottir, Einar Bjornsson, Alfons Ramel

**Affiliations:** 1 Unit for Nutrition Research, Landspitali, The National University Hospital of Iceland and Faculty of Food Science and Nutrition, School of Health Sciences, University of Iceland, Reykjavik, Iceland; 2 Department of Gastrointestinal Diseases, Landspitali, The National University Hospital of Iceland and Faculty of Medicine, School of Health Sciences, Reykjavik, Iceland

**Keywords:** Inflammatory bowel disease, Dietary intake, Nutritional status

## Abstract

**Background and aims:**

Inflammatory bowel disease (IBD) can be associated with nutritional problems. The aim of this study was to investigate diet and nutritional status of IBD patients.

**Methods:**

A total of 78 participants (35 men and 43 women aged 18–74 years) were included in this cross-sectional study. The majority (80 %) of the participant received infliximab treatment. Participants filled out disease related questionnaires and 31 participants also a 3-day food record. Body composition was measured and blood samples analysed in order to estimate nutritional status.

**Results:**

The majority (87 %) claimed that diet affects digestive tract symptoms and 72 % had changed diet accordingly. The most common foods restricted were dairy products (60 %), processed meat (55 %), soft drinks (46 %), alcohol (45 %) and fast food (44 %). Body mass index was mostly in the overweight range but 46 % of the participants had been diagnosed with some nutritional deficiency since IBD diagnosis (most common was iron deficiency: 39 %). Patients who restricted meat products had lower ferritin values (48 ± 39 vs. 95 ± 74 μg/L, *P* = 0.011). Intake of vitamin D and calcium were not adequate (65 % below recommeded intake for both) and 60 % had poor vitamin D status.

**Conclusion:**

IBD patients often change their dietary intake in order to affect digestive tract symptoms. Many patients have a history of nutrient deficiency. Restriction of dairy and meat consumption is common and is negatively associated with intake or status of micronutrients like calcium and iron. Dietary advice by a dietitian and use of potentially helpful dietary supplements is indicated.

## Background

Patients with Inflammatory bowel disease (IBD), both with Crohn’s disease (CD) and ulcerative colitis (UC), have abdominal symptoms that can both affect the intake and absorption of nutrients [1]. After diagnosis of IBD the disease process can lead to many nutritional challenges, both during remission and relapses. Decreased dietary intake, nutrient malabsorption, macro- or micronutrient deficiencies, weight loss and osteoporosis are some of the nutritional challenges people have to face [2, 3].

It is not uncommon that patients associate certain foods or food groups with exacerbation of symptoms in their digestive tract and therefore avoid certain types of foods and beverages. As a consequence, studies have been conducted to find out what food types are related to symptoms in patients [4, 5]. However, it has been difficult to generalize findings, because some food items reported to be beneficial for one patient can be detrimental to another patient [4]. Thus, it is rarely possible to identify a specific group of food items that should be avoided by all patients and thus a personalized diet is important in the management of these individuals [6].

As patients frequently avoid certain food groups, patients might be at risk of insufficient dietary intake. Furthermore, some of patients with IBD, particularly those from Crohn’s disease even suffer from nutritional deficiencies especially associated with malabsorption. Therefore, not surprisingly, micronutrient deficiency in IBD is common and well documented for iron, folic acid and vitamin B12 [7]. Iron deficiency is considered the most common micronutrient deficiency, reported in up to 39 % of IBD patients [8] and with up to 65 % of patients requiring iron replacement over the course of their disease [9]. Vitamin B12 deficiency appears to be common in patients with ileal CD or resection of the ileum [10] and prevalence of subnormal levels of vitamin B12 has been reported in 18 % of IBD patients [8].

Restriction of dairy products seems to be quite common among IBD patients or up to 65 % [11]. As a possible consequence, inadequate calcium intake has been reported in one third of patients [12] along with inadequate intake of vitamin D (36 %) [8] and low serum vitamin D concentrations [13]. This can be clinically relevant as osteoporosis has been reported among these patients [14–18] and fracture among patients with IBD is 40 % greater than in general population [19].

In order to gain more knowledge on IBD and diet, the aim of the present study was to investigate dietary intake, food preferences and nutritional status of Crohn’s disease and ulcerative colitis patients in Iceland.

## Material and methods

### Participants and study design

This cross-sectional study included 78 patients (35 men and 43 women aged 18–74 years) with the diagnosis of either Crohn’s disease or ulcerative colitis. The data collection was carried out from April 2013 to January 2014. The participants were recruited by advertisements from all over Iceland but most of them were from the Reykjavik capital area. The sample size was not based on sample size/power calculations, but rather presents the number of available patients in the Reykjavik area willing to participate. Most of the participants (*n* = 62) were on treatment with infliximab therapy receiving infusion every five to eight weeks. The rest of the participants (*n* = 16) used other IBD drugs or no medicines at all. The vast majority of patients were in clinical remission, as assessed by their responsible physician.

### Ethics, consent and permissions

The study was approved by the Icelandic National Bioethics Committee (VSN-2013020067/0307) and all persons gave their informed consent prior to their inclusion in the study.

### Questionnaire

Participants answered a questionnaire which contained 65 questions about the disease, symptoms, medications, allergies, supplementations, diet and food preferences.

### Body composition measures

Body weight was measured on a calibrated scale (model no. 708, Seca, Hamburg, Germany) and height was measured with a calibrated stadiometer (model no. 206; Seca, Hamburg, Germany). Body mass index (BMI) was calculated from the recorded height and weight. Waist circumference was measured halfway between the top of the lateral iliac crest and the lowest rib. Body fat% was assessed by bioelectrical impedance analysis (Body Fat Monitor BF 306, Omron Healthcare UK Ltd, Milton Keynes, United Kingdom).

### Blood samples

Blood samples were taken from 62 patients by the nurses in the hospital prior to the infusion of infliximab. The blood values obtained in the present study were the following: hemoglobin, albumin, C-reactive protein (CRP), iron, total iron binding capacity (TIBC), ferritin, vitamin B_12_, folate and 25-OH vitamin D (25OHD). Blood samples were not available from patients that used other IBD drugs than infliximab or no medicines at all.

### Dietary assessment

The participants recorded intake of all food, drinks and dietary supplements during three days including one weekend day (*n* = 31). The data was transferred into a nutrient calculation program, ICEFOOD version 2.0 which is based on the Icelandic nutrient composition database ISGEM. The average intake of three days for each participant was calculated. The authors want to comment here that a 7 day food record would have been more preferable, however, experience has hown that even a 3 day record is a considerable threshold for subjects to participate.

### Data analysis

Statistical analyses were performed using SPSS 20 (SPSS Inc., Chicago, IL, USA). Data are described as mean ± standard deviation (SD) or as median and inter-quartile-range (IQR). Data were checked for normal distribution using the Kolmogorov-Smirnov test. An independent samples *t*-test (normal distribution) or a Mann-Whitney *U* test (non-normal distribution) was used to assess the difference between two groups. The level of significance in the study was *P* < 0.05.

## Results

A total of 78 patients (35 men and 43 women aged 18–74 years) with the diagnosis of either Crohn’s disease (55 %) or ulcerative colitis (45 %) were recruited from the outpatient clinic and participated in the study. Table 1 shows the characteristics of the participants. The mean BMI for both genders was in the overweight range, only one man (2.9 %) and one women (2.3 %) were with a BMI below 18.5 kg/m^2^. The majority of the participants had changed their diet during the course of the disease and also the majority claimed that diet affects digestive tract symptoms. Foods and beverages most frequently claimed to have negative effects on symptoms and thus avoided were dairy products (60 %), processed meat (55 %), soft drinks (46 %), alcohol (45 %), fast food (44 %), spicy food (41 %), citrus fruits (41 %), cabbage (26 %), meat (26 %) and coffee/tea (36 %). Foods that were mentioned to have positive effects on symptoms were fish (22 %), non-processed food (8 %), chicken (6 %), and nutritional drinks (6 %). Fruits and vegetables were both mentioned to have negative as well as positive effects but slightly more people found them negative or 16.5 % vs. 13.5 %.

**Table 1:** Characteristics of the participants

	All (*N* = 78)	Males (*n* = 35)	Females (*n* = 43)
Age	40 ± 12.7	39 ± 10.8	41 ± 14.0
Height (cm)	173 ± 8.7	179 ± 6.9	168 ± 5.9^a^
Body Weight (kg)	79.2 ± 16.4	82.1 ± 16.1	76.9 ± 16.5
BMI (kg/m^2^)	26.5 ± 5.4	25.5 ± 4.6	27.4 ± 5.9
Waist (cm)	92.6 ± 13.3	93.3 ± 11.6	92.2 ± 14.6
Body fat (%)	29.4 ± 9.9	22.4 ± 7.8	36.2 ± 6.4
Crohn’s disease	43 (55 %)	22 (63 %)	21 (49 %)
Ulcerative Colitis	35 (45 %)	13 (37 %)	22 (51 %)
Infliximab/Adalimumab	65 (83 %)	35 (100 %)	30 (70 %)^a^
Have met a dietitian	32 (41 %)	13 (37 %)	19 (44 %)
Smokers	16 (21 %)	9 (26 %)	7 (16 %)

Data are presented as mean ± SD or as N and % of all, males and females

*Abbreviations*: *BMI* body mass index, *SD* standard deviation

^a^significantly different from male participants

The use of dietary supplements among participants is shown in Table 2. Cod liver oil and vitamin D supplements were the most commonly used supplements. Although 47 patients (60 %) reported to consume less or even no dairy products, but only 8 of them used calcium supplements. Dietary calcium intake of 72 % of those who restrict dairy products did not reach the recommended daily intake of 800 mg/d.

**Table 2:** Current intake of supplements among participants

	All (*N* = 78)	Male (*n* = 35)	Female (*n* = 43)
Cod liver oil	48 (62 %)	23 (66 %)	25 (58 %)
Vitamin D	32 (41 %)	11 (31 %)	21 (49 %)
Multivitamin	21 (27 %)	6 (17 %)	15 (35 %)
Vitamin B-12	14 (18 %)	5 (14 %)	9 (21 %)
Calcium	12 (15 %)	3 (9 %)	9 (21 %)
Iron	12 (15 %)	2 (6 %)	10 (23 %)^a^
Probiotics	10 (13 %)	3 (9 %)	7 (16 %)

Data are presented as N and % of all, males and females

^a^significantly different from male participants

Almost half of the participants (46 %) had been diagnosed with a nutritional deficiency after their diagnosis of CD or UC. Iron deficiency was the most frequent deficiency (39 %) which was more frequent in women younger than 50 years than in women older than 50 years or in men (58 vs. 25 % vs. 26 %, respectively, *P* = 0.015). Previous diagnose of deficiency of vitamin B_12_ (17 %), vitamin D (4 %) and folic acid (1 %) were less common.

Table 3 shows the participants’ dietary intake (from food and dietary supplements) in comparison to the Nordic Nutrition Recommendations 2012 [20] and to results from the Icelandic National Dietary Survey from 2011 [21]. Analysis of the micronutrient and vitamin intake shows that intake was below recommended levels, especially true for women. e.g., 65 % of the participants had a calcium intake below 800 mg/d and 16 % even below 400 mg/d. When the daily intake of vitamin D was examined we found out that 65 % did not reach the Icelandic recommendations and 29 % were even below 2.5 μg/d, which is the lower level of intake for vitamin D. Seventy-seven% of women did not reach the recommended daily intake of iron. Vitamin B_12_ intake however was sufficient, but participants who reduced intake of milk products had less intake of vitamin B_12_ (4.7 ± 3.0 vs. 9.4 ± 8.2 μg B_12_/day, *P* = 0.042).

**Table 3:** Mean intake (food and supplements) of macronutrients and median intake of dairy products, calcium, vitamin D, iron and vitamin B_12_ per day

(*n* = 31)	mean ± SD		%E	Recomended %E^a^	National survey 2012^b^
Energy (kcal)	1860 ± 715				2059 kcal
Protein (g)	87 ± 36		19 %	10–20 %	93 g (18 %)
Fat (g)	73 ± 33		35 %	25–40 %	83 g (36 %)
Carbohydrates (g)	195 ± 82		42 %	45–60 %	216 g (42 %)
Fibre (g)	17.5 ± 6.7		2 %	23 g	17 g (1.7 %)
Added sugar (g)	38.5 ± 34.7		8 %	>10 %	47 g (9 %)
	median (IQR)	Male (*n* = 14)	Female (*n* = 17)	RDI^a^	mean ± SD
Dairy products (g)	190 (232)	218 (550)	133 (95.3)	–	300 ± 232
Calcium (mg)	717 (302)	877 (718)	674 (204)	800	923 ± 428
Vitamin D (μg)	6.5 (18.6)	13.1 (17.3)	4.3 (13.2)	10/15^b^	8.1 ± 9.3
Iron (mg)	10.8 (8.4)	11.5 (10)	8.5 (7.5)	9^c^/15^d^	10.9 ± 5.8
Vitamin B_12_ (μg)	5.1 (5.7)	7.2 (6.7)	4.3 (4.8)	2.0	6.9 ± 8.5

*Abbreviations*: *SD* standard deviation, *%E* % of total energy, *RDI* recommended daily intake

^a^According to Nordic Nutrition Recommendation 2012

^b^According to the Icelandic National Dietary Survey 2010–2011

^c^Male

^d^Female

In Table 4, the results of blood samples of the participants are demonstrated. Women were more often below reference values in various iron indices (iron: 22 % vs. 3 %, *P* =0.06; ferritin: 18.5 % vs. 0 %, *P* = 0.009). Participants who reduced meat intake in course of their disease had lower blood values of ferritin compared to others (47.5 ± 38.6 vs. 95.1 ± 73.5, *P* = 0.011). This difference was still significant after correction of age and gender.

**Table 4:** Results of blood samples of the participants

	All (*N* = 64)	Male (*n* = 35)	Female (*n* = 29)	Reference ranges
Hemoglobin (g/L)	139 ± 14.2	148 ± 9.5	128 ± 11.3^a^	134–171^b^/118–152^c^
Albumin (g/L)	41 ± 3.7	42 ± 3.3	40 ± 3.8^a^	36–48
CRP (mg/L)	8.3 ± 7.8	6.9 ± 5.4	10 ± 9.8	<10
Iron (μmol/L)	17.9 ± 8.1	19.6 ± 7.8	15.7 ± 8.1	9–34
TIBC (μmol/L)	61.3 ± 14.3	58.3 ± 15	64.9 ± 12.9	49–83
Ferritin (μg/L)	81.9 ± 68.8	100 ± 66.7	58.4 ± 65.2^a^	30–400^b^/15–150^c^
Vitamin B_12_ (pmol/L)	385 ± 133.8	385 ± 134	384 ± 137	210–800
Folate (nmol/L)	23 ± 10.3	23.4 ± 10	22.6 ± 10.6	6–35
Vitamin D^d^ (nmol/L)	51.9 ± 32.2	47.5 ± 33	57.5 ± 30.4	50–150

Data are presented as mean and SD

*Abbreviations*: *SD* standard deviation, *CRP* C-reactive protein, *TIBC* total iron binding capacity

^a^significantly different from male participants

^b^Male

^c^Female

^d^25OHD

Although only the minority of participants had received a diagnosis of vitamin D deficiency previously (see above), the measured 25OHD concentrations were low and 60 % of the participants were below 50 nmol/L and 26 % even below 30 nmol/L. Participants taking vitamin D supplements had as might be expected higher levels of vitamin D than those not taking any vitamin D supplements (70.6 ± 40.5 vs. 41.8 ± 21.2 nmol/L, *P* = 0.007). There was not a clear association between 25OHD status and weeks of the year and no association between intake of cod liver oil and 25OHD status (data not shown). Similarly,patients who recieved vitamin B_12_ supplements had higher vitamin B_12_ blood values those who did not (505 ± 215 vs. 361 ± 99 pmol/L, *P* = 0.001).

## Discussion

The results of the current study showed that patients associate certain food groups to digestive tract symptoms. The vast majority of the participants (87 %) claimed that diet can affect these symptoms and 72 % have changed their diet accordingly after they were diagnosed with IBD. The most common food groups mentioned to worsen symptoms were dairy products, processed meat, fast food, soft drinks, alcoholic beverages but also citrus fruits and cabbage. However, due to inter-individual variability, i.e., some foods are mentioned as having both negative as well as positive effects on the disease. Thus it is very difficult to give general dietary advice for this patient group.

Body composition can change as a consequence of IBD. Thus, patients with IBD should routinely have their body weight measured [22] as the prevalence of protein-energy malnutrition has been reported to be 20–85 % [7]. Interestingly, the prevalence of malnutrition according to BMI has decreased over the years as indicated by recent studies showing lower prevalence rates in IBD patients [23] which may be related to improved therapy that can induce disease remission and keep the patients in remission. Most patients in the present study had BMI even in the overweight range. The reported mean intakes of macronutrients (E%) were comparable to the results from the Icelandic National Dietary Survey [21] and in line with the Nordic nutrition recommendations 2012 [20], with the exceptions of carbohydrates, which deliver less than 45 % of energy, and fibre. Although energy intake of the participants seemed to be satisfying, both in terms of BMI and estimated intake of macronutrients, nearly half of the participants (46 %) have been diagnosed with some nutritional deficiency during their history of IBD. In clinical practice, micronutrient deficiency in IBD is common but in most cases it does not tend to have any clinical manifestation except with regard to iron, folic acid and vitamin B12 [7].

In general, patients with IBD are at greater risk of developing metabolic bone disease as high prevalence of osteoporosis has been reported among these patients [14–17]. In the current study more than half of the participants restricted their intake of dairy products which is similar to recently published findings from Brasil-Lopes et al. [11]. Thus, not surprisingly, dietary calcium intake was found to be inadequate in this group and 72 % did not achieve recommended intake. Unfortunately, only 15 % of those who restrict their dairy intake took calcium supplements.

Another important nutrient to ensure good bone health is vitamin D. An intervention study showed that use of calcium and vitamin D supplements has positive effects on bone health in IBD [24]. Forty-one percent of our participants used vitamin D supplements and these individuals have significantly higher 25OHD concentrations than those who do not take supplements. Even more frequently (62 %) cod liver oil was used on a regular basis. This is higher than the numbers from the Icelandic National Dietary Survey [21], according to which 43 % of adults in Iceland take cod liver oil [21]. However, in our study there was no significant association between intake of cod liver oil and 25OHD status indicating that amount and/or frequency of cod liver oil used are not always sufficient. Inadequate uptake of micronutrients from the intestinal tract can occur in IBD patients but mostly in those with active disease [25]. However, the vast majority of the patients in the present study were in clinical and biochemical remission.

Nearly two thirds of the patients had 25OHD below the threshold of 50 nmol/L. Considering previous Icelandic studies on vitamin D, it shows that in our participants there was a similar or somewhat higher prevalence of inadequate 25OHD (60 %) as compared to community dwelling old adults (41–50 %) [26], geriatric hospitalized patients (56 %) [27] and young adults (61 %) [28]. Considering this low intake of dairy products along with low 25OHD levels, more supervision and education/guidance to the patients seems to be of importance in order to reduce the risk osteoporosis can be considered as beneficial.

Iron deficiency had been detected in 39 % of the participants (58 % in women younger than 50 years) since the diagnosis of IBD. This is in accordance with previous studies which reported iron deficiency the most common micronutrient deficiency [8] and with up to 65 % of patients requiring iron replacement over the course of their disease [9]. The associated anemia is clinically important and can affect quality of life [25]. Despite the high prevalence of iron deficiency in IBD, only 15 % of our participants were on iron supplements. Interestingly, iron intake was not significantly different between patients who restricted meat and meat products in comparison with those who did not. However, ferritin values were significantly lower in those who did not eat meat. It is well known that iron bioavailability from meat is usually better than from plant sources [29].

Dietary intake of vitamin B12 was high both for men and women. The participants who reduced intake of milk products had lower intake of vitamin B12 than those who did not reduce milk intake but still higher than the recommended intake. This can be partly explained by the fact that milk products contribute to vitamin B12 intake and according the latest national dietary survey in Iceland this contribution is 15 % [19]. Mean vitamin B12 levels in blood were well above the recommended minimum.

Dietary supplements have been discussed or suggested for IBD patients in order to make up for a deficiency or to prevent a deficiency from occurring. Supplements that may be needed include among others calcium, vitamin D, iron, vitamin B12 and folate. However, before taking dietary supplements, several issues have to be considered. Vitamin and mineral supplements can cause GI symptoms. Supplements can contain lactose, artificial colors, sugar alcohol or preservatives which IBD patients can react to. It is thus indicated to discuss all dietary supplements with members of the health care team [30].

### Limitations

This study was of cross-sectional nature and thus cannot differentiate between cause and effect in an observed association. Further on, we used subjective information from patients, e.g., food groups and digestive tract symptoms, which cannot be considered proof for a causal relation between e.g., milk and symptoms. Rather we used this information to detect potential nutrition related problems, e.g., poor calcium intake, derived from avoidance of certain food groups. Also, this is a selected group of IBD patients, mostly on biological treatment and most in clinical remission. Therefore we can not extrapolate the results of or study into an unselected group of IBD patients and not on those with active disease, who probably are more affected by nutritional deficiency.

## Conclusion

Our study showed that IBD patients often change their dietary intake in order to affect digestive tract symptoms. Many patients have a history of nutrient deficiency. Restriction of dairy and meat intake was common and can negatively influence intakes or status of micronutrients like calcium and iron. Dietary advice by a dietitian and use of potentionally helpful dietary supplements is often indicated.
